# Prevalence and Antimicrobial Resistance Patterns of *Escherichia coli* in the Environment, Cow Dung, and Milk of Selangor Dairy Farms

**DOI:** 10.3390/antibiotics14020137

**Published:** 2025-02-01

**Authors:** Yuvaneswary Veloo, Sakshaleni Rajendiran, Zunita Zakaria, Rohaida Ismail, Salina Abdul Rahman, Rozaihan Mansor, Syahidiah Syed Abu Thahir

**Affiliations:** 1 National Institutes of Health, Ministry of Health, Shah Alam 40170, Malaysia; sakshaleni@moh.gov.my (S.R.); rohaidadr@moh.gov.my (R.I.); sar@moh.gov.my (S.A.R.); syahidiah@moh.gov.my (S.S.A.T.); 2 Institute of Bioscience, Universiti Putra Malaysia, Serdang 43400, Malaysia; 3 Faculty Veterinary Medicine, Universiti Putra Malaysia, Serdang 43400, Malaysia; rozaihan@upm.edu.my

**Keywords:** antimicrobials, antimicrobial resistance, dairy farm, environment, milk

## Abstract

Background/Objectives: The increasing threat of antimicrobial resistance (AMR) to global public health urgently needs attention. Misuse of antimicrobials in sectors such as dairy farming has led to the emergence and spread of resistant bacteria and genes. This study investigated AMR patterns and profiles of *Escherichia coli* (*E. coli*) from various sources, including soil, effluent, cow dung, and milk. Methods: A total of 192 samples were collected, comprising environmental samples (soil and effluent), cow dung samples, and milk samples from eight dairy farms in Selangor, Malaysia. The spread plate method was employed to isolate *E. coli,* and all the isolates were subjected to Gram staining to identify Gram-negative, rod-shaped bacteria. The Vitek^®^ 2 system was used for *E. coli* identification and susceptibility testing. Results: The prevalence of *E. coli* identified in the eight farms was 66.1%. A total of 360 *E. coli* isolates were successfully isolated, and 19.7% of the isolates presented AMR with ampicillin exhibiting the highest resistance (18.3%), followed by trimethoprim–sulfamethoxazole (8.9%). Additionally, 8.9% of them were multidrug resistant, which could be divided into 16 patterns. For the extended spectrum beta-lactamase screening, nine isolates were positive. Conclusions: This finding emphasizes the rise in resistant isolates in the growing dairy industry and underscores the urgency of addressing the potential reservoir of AMR. Therefore, essential measures such as continuous surveillance and effective antimicrobial stewardship programs are crucial for regulating veterinary antimicrobial use. Research on the mechanisms driving the development and dissemination of AMR is imperative for addressing One Health concerns.

## 1. Introduction

Antimicrobial resistance (AMR) represents one of the greatest threats to global public health [1]. AMR poses serious risks to human well-being, leading to prolonged illnesses, increased morbidity and mortality rates, and increased healthcare costs [2]. The burgeoning of AMR was evident in 2019, with 1.27 million global deaths attributed directly to AMR and 4.95 million deaths from illnesses related to bacterial AMR [1]. Without immediate action, projections suggest 10 million annual fatalities and USD 100 trillion in economic losses globally by 2050 [1,3]. The acceleration of the AMR crisis is closely linked to the widespread abuse and overuse of antimicrobials worldwide. Inappropriate prescribing practices and extensive use across various sectors, notably agriculture, have been the primary drivers of AMR development and spread [1,4,5].

Dairy farming represents a significant sector contributing to antimicrobial use in agriculture [6]. With an increasing demand for milk and dairy products, worldwide dairy production is projected to increase rapidly, reaching 977 million tons by 2029 [7]. In Southeast Asia, including Malaysia, smallholder dairying holds great potential for a highly profitable and sustainable industry [8,9,10]. Corresponding to this growth, antimicrobial use in the dairy industry is also on the rise [6,11]. These substances are primarily employed to treat and prevent infections in dairy cows, such as mastitis, lameness, respiratory illness, and enteric diseases, as well as to enhance growth and feed efficiency [6,11]. Consequently, food safety and the quality of dairy milk and products have become major concerns [10].

The excessive and improper use of antimicrobials in dairy farms has led to the emergence and spread of antimicrobial-resistant bacteria (ARB) and antimicrobial resistance genes (ARGs). These resistant pathogens and genes can be transferred through various pathways, threatening human and animal health, the environment, and the food chain [12,13]. Antimicrobial residues, ARBs, and ARGs are often found in animal waste, contaminating the soil environment and reaching nearby water sources [14,15,16]. Furthermore, they can be introduced into the food chain at any point during the farm-to-form scale [17]. These findings underscore the health risk associated with AMR.

Among the multidrug-resistant pathogens highly prevalent on dairy farms, *Escherichia coli* (*E. coli*) is particularly concerning, as certain strains can cause foodborne infections in humans [18] and are among the most commonly reported mastitis-causing pathogens in dairy farms [19]. As a zoonotic bacterium in nature, *E. coli* colonizes the digestive systems of cattle and other animals [20]. *E. coli* serves as an indicator organism for AMR surveillance and acts as a reservoir for ARGs, which can be transferred to animals and humans through numerous pathways, such as direct animal contact, contaminated food, water sources, and farm environments [18,21]. Therefore, monitoring commensal *E. coli* from dairy farms to understand AMR patterns and profiles is essential [21,22]. At present, most of the studies on antimicrobial-resistant *E. coli* are in the fields of human and animal medicine, while data concerning the environment and food products are very scarce.

The aim of this study was to determine the prevalence of *E. coli* and to determine AMR patterns of the *E. coli* strains isolated from various samples, including the environment, cow dung, and milk from dairy farms across Selangor State, Malaysia. The results from this study can serve as a surveillance tool to guide antimicrobial stewardship, and they are essential for promoting sustainable agricultural practices and protecting both animal and human health from the threat of AMR.

## 2. Results

### 2.1. Prevalence of E. coli in Dairy Farms

The prevalence of *E. coli* from the eight dairy farms was 66.1% (127/192), as shown in Table 1. An in-depth analysis then revealed that the prevalence of *E. coli* was 58.3% in soil samples, 64.6% in effluent, 85.4% in cow dung, and 56.3% in milk samples. A total of 360 *E. coli* isolates were identified from eight dairy farms in Selangor. Among the *E. coli* isolates, 61 (16.9%) were from soil, 88 (24.4%) were from effluent, 141 (39.2%) were from cow dung, and 70 (19.4%) were from milk samples. An identification test using the VITEK^®^2 system (bioMérieux, Nurtingen, Germany), also revealed that one *E. coli* isolate was classified as *E. coli* O157:H7. This isolate was sourced from a milk sample collected at Farm 4.

### 2.2. Antimicrobial Resistance Rate in E. coli

**Figure 1:**
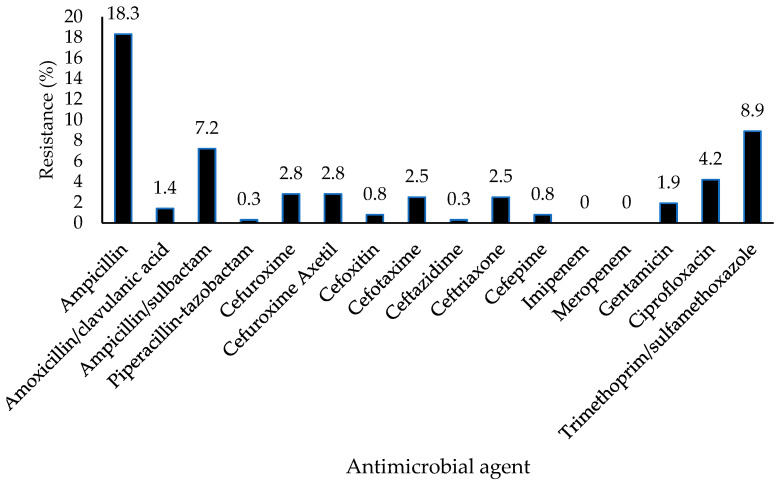
The percentage of resistance of *E. coli* based on antimicrobial agents.

**Figure 2:**
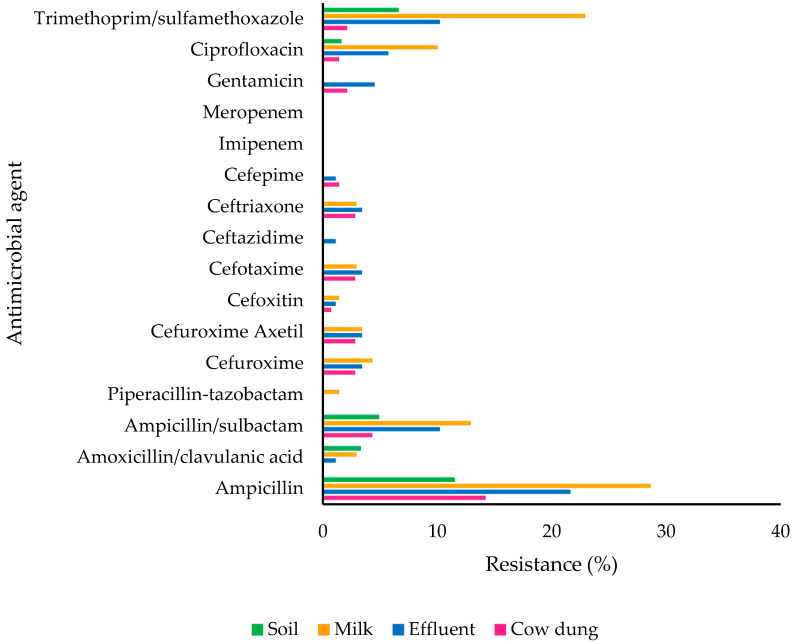
Percentage of resistance of *E. coli* to antimicrobial agents based on the types of samples.

**Figure 3:**
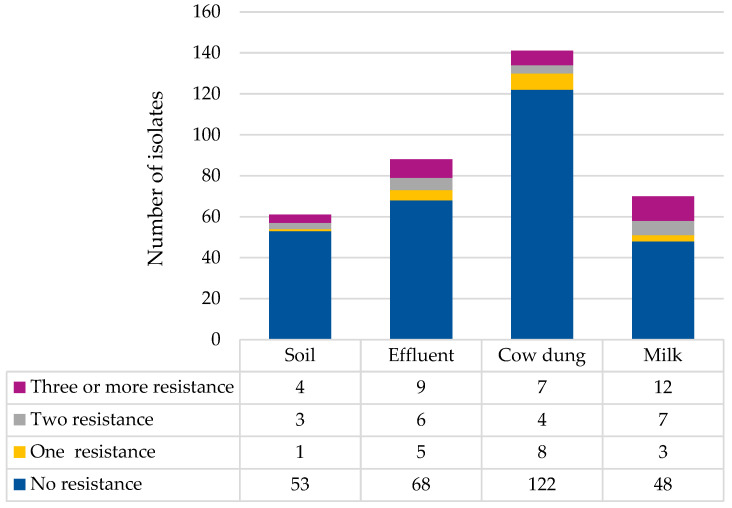
AMR patterns of *E. coli* based on sample types.

**Figure 4:**
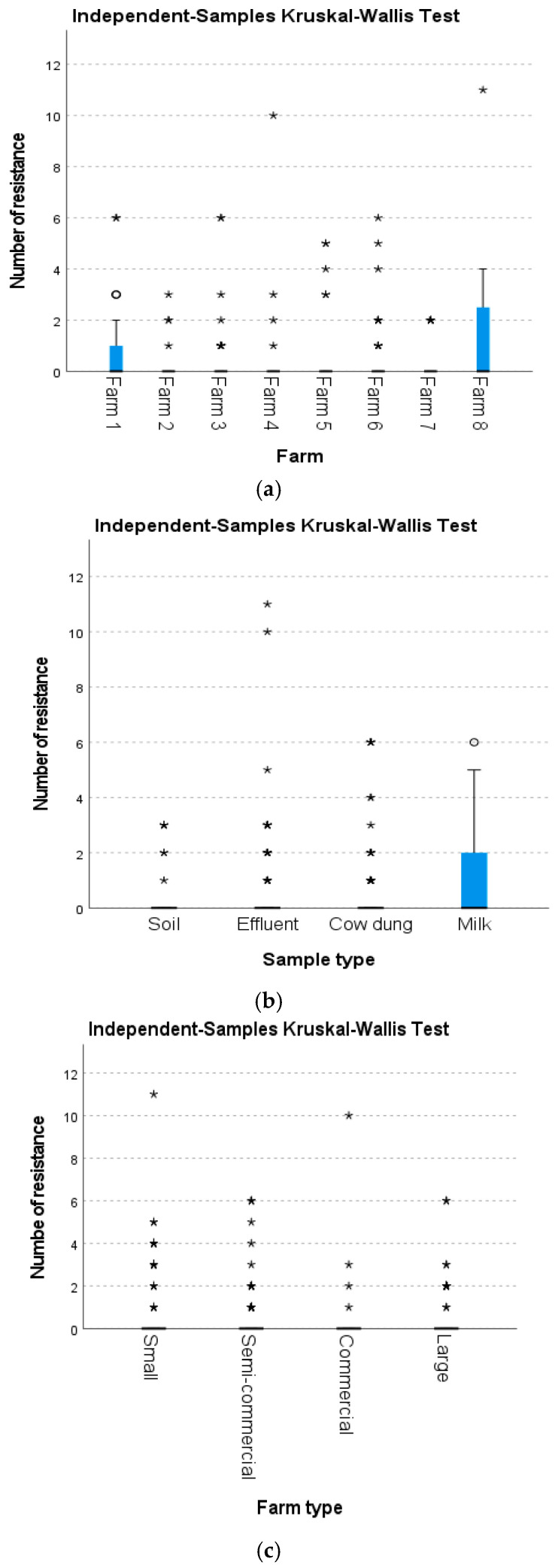
(**a**) Kruskal–Wallis test to compare resistance patterns based on the eight farms; (**b**) Kruskal–Wallis test to compare resistance patterns based on the sample types; and (**c**) Kruskal–Wallis test to compare resistance patterns based on the farm types. The asterisks (*) in the plot represent individual data points. The circle (o) represents an outlier in the data.

The isolates were subjected to antimicrobial susceptibility testing. Overall, 19.7% (71/360) of the isolates exhibited resistance to at least one antimicrobial agent. Notably, ampicillin had the highest resistance rate (18.3%), followed by trimethoprim–sulfamethoxazole (8.9%) and ampicillin–sulbactam (7.2%) (Figure 1). None of the isolates exhibited resistance to imipenem or meropenem, both of which are carbapenems. Figure 2 shows the number of isolates with resistance to antimicrobial agents categorized based on the types of samples. The results showed that 13.1% (*n* = 8/61) of *E. coli* isolated from soil, 22.7% (*n* = 20/88) from effluent, 13.5% (*n* = 19/141) from cow dung, and 31.4% (*n* = 22/70) from milk exhibited resistance to at least one antimicrobial agent (Figure 3). To evaluate the differences between farms, types of farms, and types of samples with resistance patterns, the Kruskal–Wallis test was utilized as the data were not normally distributed. The Kruskal–Wallis test revealed a significant difference in the resistance pattern of *E. coli* between farms and sample types (*p* < 0.05). Nonetheless, no significant difference was observed in the resistance pattern of *E. coli* among farm types (*p* > 0.05) (Figure 4a–c).

### 2.3. Multidrug Resistance (MDR) Profiles in E. coli

Based on the results, 8.9% (32/360) of isolates were resistant to three or more antimicrobials categories. Furthermore, 28.1% (9/32) were from effluent, 37.5% (12/32) from milk, 21.9% (7/32) from cow dung, and 12.5% (4/32) from soil samples. For MDR profiling, multidrug-resistant *E. coli* can be divided into 16 patterns, as shown in Table 2.

### 2.4. ESBL-Producing E. coli

Nine (2.5%) isolates were identified as ESBL positive, of which 2.8% (4/141) originated from cow dung, 4.3% (3/70) from milk, and 4.3% (2/88) from effluent. All isolates also exhibited MDR. The findings indicated that one of the ESBL-producing isolates from effluent demonstrated 62.5% (10/16) AMR, one of the ESBL-producing isolates from cow dung demonstrated 43.8% (7/10) AMR, and the rest of the ESBL-producing cow dung isolates demonstrated 37.5% AMR each. The details on the AMR of these ESBL-producing isolates are shown in Table 3.

## 3. Discussion

The main aim of this study was to determine the prevalence and AMR patterns of *E. coli* from various sources in dairy farms, including the environment (soil and effluent), cow dung, and milk. Currently, very limited data are available in Malaysia and other countries pertaining to AMR on dairy farms, especially concerning environmental components.

This study identified *E. coli* in 58.3%, 64.6%, 85.4%, and 56.3% of soil, effluent, cow dung, and milk samples, respectively. The findings were aligned with the outcomes of other reported studies. For example, a study in Punjab, India, reported *E. coli* in 60% of the slurry samples, and a study in Indonesia revealed that 69.3% of the samples were positive for *E. coli* [23,24]. In Malaysia, a study on bacteriological quality and safety of raw milk revealed 65% were *E. coli* positive [25]. However, a study in Holeta district, Central Ethiopia, reported a lower prevalence of 19% for *E. coli* in raw milk samples [26]. Variations in the prevalence of *E. coli* may be influenced by geographical locations, farming practices, and sanitation standards. Moreover, the high prevalence of *E. coli* in milk indicates poor hygienic practices during milking and handling, potentially resulting in direct or indirect fecal contamination while posing a risk to consumers of contaminated milk [27].

In this study, only one isolate from milk sample was identified as *E. coli* 0157:H7, while the remaining isolates were identified as *E. coli* via the Vitek^®^ 2 system. Despite the isolation and identification of 360 *E. coli* isolates from environmental, cow dung, and milk samples, only one isolate (0.3%) tested positive for *E. coli* 0157:H7. This finding is supported by a study in Malaysia that reported a low presence (3.6%) of *E. coli* 0157:H7 isolates in cattle, environments, milk, and beef, suggesting the potential absence of this isolate in cattle farms [28]. However, these findings contrast slightly with those of other studies, such as that of Ariyanti et al., where 15.6% of *E. coli* 0157:H7 were identified in milk samples in Indonesia [29]. Similarly, Mesele et al. reported a 4.7% prevalence of *E. coli* 0157:H7 in various samples [28]. These findings suggest that cattle in Malaysia are not significant reservoir for *E. coli* serotype 0157:H7. However, its presence in raw milk highlights the potential public health risk.

In this study, resistance in *E. coli* was highest against ampicillin (18.3%), followed by trimethoprim–sulfamethoxazole (8.9%), and ampicillin–sulbactam (7.2%). Meropenem and imipenem were proven to be effective against *E. coli.* Resistant *E. coli* was highly present in milk (31.4%) and effluent (22.7%). According to the Malaysia National AMR Data Surveillance (2018–2021), *E. coli* is 100% resistant to erythromycin and 53% resistant to ampicillin, whereas it is 100% susceptible to gentamicin [30]. The percentage of resistance to ampicillin in this study is consistent with data from Kim et al. and Huang et al. [31,32], although it was found to be higher in most of the studies [33,34,35,36,37].

A report by Hinthong et al. [38] in Thailand revealed that the percentage of penicillin-resistant *E. coli* (92.2%) was the highest, followed by resistance in the folate pathway-inhibitor category (26%), and the Cephems category (18.2%). Similarly, most studies have shown high susceptibility (96.6–100%) to meropenem and imipenem [35,38,39,40]. This finding is understandable because ampicillin is often used in livestock farms to treat mastitis and endometritis in dairy cows [23,31]. The elevated resistance of trimethoprim–sulfamethoxazole and ampicillin–sulbactam is attributed to the extensive use of this combination due to its broad-spectrum activity, and effectiveness against infections [23,41,42]. According to Hassali et al., in Malaysia, 66.6% of the antimicrobial products registered by the National Pharmaceutical Regulatory Agency (NPRA) were for use in livestock, with 45 products under the β-lactam group of drugs and 43 under the combination drug containing trimethoprim [43].

Based on these findings, even though the percentage of resistant *E. coli* varied between samples, resistance to similar types of antimicrobials was commonly detected. For example, a high percentage of resistance was observed to ampicillin, trimethoprim–sulfamethoxazole, and ampicillin–sulbactam across all the samples, whereas 100% susceptibility was observed for imipenem and meropenem. This highlights the potential transmission route of antimicrobial-resistant *E. coli* between different sources, including the environment, food, and animal waste [44,45]. Furthermore, the consistent detection of resistant *E. coli* across sample types suggests that such bacteria may spread through more interconnected pathways [44,45]. Therefore, a better understanding of these transmission dynamics is crucial for developing comprehensive strategies to mitigate the spread of AMR.

The occurrence of ESBL-producing *E. coli* was considered very low in this study (2.07%), similar to the findings of Kamaruzzaman et al., who reported that 4.8% of dairy farms in Malaysia were ESBL-producing *E. coli*, with 0.27% in feces, 1.32% in farm environments, and 3.18% in milk samples [46]. Consistent with these findings, the resistance of *E. coli* in this study was low towards third- and fourth-generation cephalosporins, such as cefotaxime (2.1%), ceftazidime (0.3%), ceftriaxone (2.1%), and cefepime (0.9%), respectively. In contrast, Liu et al. reported that 70.59% (12/17) of *E. coli* were resistant to β-lactams in their study, and another study in Assam, India, reported a 35% prevalence of ESBL-producing *E. coli* in cow dung samples [35,47]. These findings suggest that limited use of β-lactam agents for treating mastitis and prophylactic may be more restrictive in dairy farms. Nonetheless, the presence of ESBL-producing *E. coli* in milk warrants increased attention due to the production of dairy products. Food-borne outbreaks associated with milk and dairy products have resulted in hospitalizations and fatalities worldwide [48].

Similarly, the percentage of MDR bacteria observed in this study was 8.9%, with MDR profiles ranging from resistance to three to eleven antimicrobials. This diversity in the AMR spectrum among isolates was evident [49]. This finding is comparable with other studies examining *E. coli* isolates from milk and environmental samples on dairy farms elsewhere. For examples, Ngaywa et al. reported 2% (6/304) MDR in *E. coli* isolates from raw milk samples [50], and Widodo et al. reported 11.7% (14/139) MDR in wastewater [51]. These percentages, however, were lower than the 21% (45/214) in milk samples reported by Mwasinga et al. [52] and the 44.4% (126/284) in various milk and environmental samples reported by Shoaib et al. [40]. The observed MDR level in this study could potentially be linked to the farmer’s practice, which involves DVS for continuous monitoring of the cattle’s heath and conditions of the studied areas, including probability of lower antimicrobial usage. Nevertheless, these findings underscore the emergence of resistant isolates and indicate the potential reservoir of AMR if proactive measures are not implemented.

### Strength and Limitations

To the best of the author’s knowledge, this study is among the first to investigate the AMR in *E. coli* from various samples, including soil, effluent, cow dung, and milk in the context of Malaysian dairy farm environments. A previous study by Kamaruzzaman et al. focused solely on ESBL-producing *E. coli* and conducted only genotypic analyses [46]. This effort is crucial for providing baseline data on *E. coli* resistance levels in environmental components, together with cow dung, which represents animals, and milk, which represents food. Furthermore, this study aligns with the Malaysian Action Plan on AMR (MyAP-AMR) and the data generated can serve as baseline database on environmental bacteria, complementing existing databases on AMR in clinical and animals’ contexts.

This study, however, possessed certain limitations that should be acknowledged. Given that this study was carried out in a specific region, the findings did not extend to other areas of the country. Additionally, this study did not include the use of questionnaires to access antimicrobial usage on farms, which could reveal a direct correlation with the AMR rates.

## 4. Materials and Methods

### 4.1. Sample Collection

**Figure 5:**
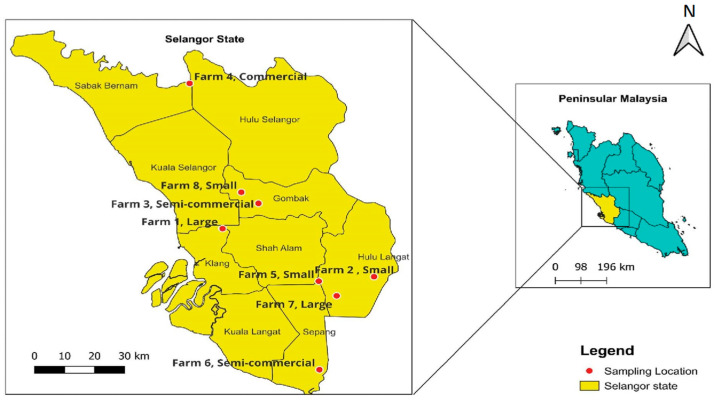
Location and scale of the selected farms in Selangor.

A cross-sectional study was carried out across eight dairy farms in the state of Selangor, Malaysia, from January 2022 to December 2023. The inclusion criteria for farm selection were that all dairy farms be registered under the Department of Veterinary Services, Selangor (DVS), and that cows be reared solely for dairying purposes, not for meat. The exclusion criteria were farms that rear other animals for milk, such as buffalo, and those not registered with the DVS. The selected farms included small (0–30 cows), semi-commercial (30–50 cows), commercial (50–100 cows), and large-scale (100 cows and above), depending on the number of lactating cows [53,54] (Figure 5).

During this study, a total of 192 samples were collected, comprising environmental samples (soil and effluent) (*n* = 96), cow dung samples (*n* = 48), and milk samples (*n* = 48).

Approximately 25 g of soil was randomly taken from various locations within each dairy farm, with distances of 10 to 20 m between sampling sites [55]. Soil samples were primarily collected near the cow barn and gracing areas. The top layer of soil, to a depth of 3 cm, was removed via a metal spade that was cleaned, disinfected with 75% alcohol, and flamed with a Bunsen burner prior to each collection [55].

Effluent samples of approximately 200 mL were collected from various locations within the farm’s drainage or water-pooled areas via a long-handled stainless-steel ladle [55]. The ladle was cleaned and disinfected similar to the metal spade, whereby it was cleaned, disinfected with 75% alcohol, and flamed with a Bunsen burner prior to each collection. The soil and effluent samples were placed in sterile zip-locked plastic bags.

Fresh cow dung samples were collected using a sterile FecalSwab^TM^ (COPAN, Jefferson Ave, Murrieta, California) containing 2 mL of Cary–Blair medium (Copan). Raw, unpasteurized cow milk samples were collected from bulk tanks via sterile disposable bottles. All samples were promptly stored in ice boxes and transported immediately to the laboratory for analysis.

### 4.2. Isolation and Enumeration of E. coli

The soil sample from the zip-lock plastic bag was manually homogenized by shaking the bag up and down before 10 g of the sample was weighed and dispensed into the first dilution Falcon tube containing 90 mL of peptone water (Difco^TM^, BD Diagnostics, Franklin Lakes, NJ, USA). For the effluent and milk samples, the plastic bags and bottles were homogenized by shaking, and 10 mL of each sample was directly transferred into the first dilution Falcon tube containing 90 mL of peptone water. Similarly, 2 mL of Cary–Blair medium containing cow dung was directly transferred into the first dilution Falcon tube containing 18 mL of peptone water. These were recorded as 10^−1^ dilutions.

The Falcon tubes were vortexed and then left to settle at room temperature. Next, the serial dilutions were continued by transferring a 1.0 mL aliquot to a new Falcon tube containing 9 mL of Difco^TM^ peptone water, followed by vortexing; this process was repeated five times (until 10^−6^). From each Falcon tube, a 1.0 mL aliquot of sample was pipetted onto the center of a commercially prepared CHROMagar™ *E. coli* (CHROMagar, Saint-Denis, France) agar plate and spread evenly throughout the plate. The plates were then incubated aerobically at 37 °C for 24 h. Three representative colonies were selected from plates containing 30 to 300 isolates for further purification via two successive subculturing steps, ensuring that pure colonies were obtained before identification and susceptibility testing were performed.

### 4.3. Identification of E. coli and Antimicrobial Susceptibility Testing

All the pure isolates were subjected to Gram staining to identify Gram-negative, rod-shaped bacteria. Identification of *E. coli*, including *E. coli* O157, was carried out via VITEK^®^2 GN (bioMérieux, Nurtingen, Germany), whereas antimicrobial susceptibility testing was performed via AST-N314 (bioMérieux, Nurtingen, Germany), following the manufacturer’s guidelines [56]. Prior to identification and testing, a bacterial suspension of each sample was prepared. Approximately 3 mL of prepared 0.45% saline (bioMérieux) was dispensed into a prelabeled, clear, 12 mm × 75 mm polystyrene test tube. Pure colonies were inoculated into saline-containing tubes and mixed well until the turbidity reached 0.50 to 0.63 McFarland via a DensiCHEK Plus instrument (bioMérieux).

The identification and testing results were interpreted on the basis of the ID-GPC database, and the final results were obtained automatically. The MIC analysis and interpretation of antibiotic susceptibility for *E. coli* was based on the Clinical Laboratory Standards Institute (CLSI) and the European Committee on Antimicrobial Susceptibility Testing (EUCAST) guidelines. The AST-N314 card comprises screening for extended-spectrum beta-lactamase (ESBL) and 16 types of antimicrobials, including ampicillin, amoxicillin-clavulanic acid, ampicillin–sulbactam, piperacillin–tazobactam, cefuroxime, cefuroxime axetil, cefoxitin, cefotaxime, ceftazidime, ceftriaxone, cefepime, imipenem, meropenem, gentamicin, ciprofloxacin, and trimethoprim–sulfamethoxazole. Quality control for VITEK^®^2 involved testing *E. coli* ATCC^®^ 25922™ and *P. aeruginosa* ATCC^®^ 27853™ in accordance with the manufacturer’s instructions, and all control minimum inhibitory concentrations (MICs) fell within the acceptable range.

### 4.4. Data Analysis

The data was analyzed via IBM SPSS Statistics version 27. The Kruskal–Wallis test was used to compare the resistance patterns of *E. coli* between farms and types of samples.

## 5. Conclusions

This study demonstrated the notable prevalence of antimicrobial-resistant *E. coli* in milk, cow dung, and dairy farm environments. The findings signified a potential public health risk concerning the possibility of extensive transmission from various sources while necessitating strategies to mitigate the spread of AMR. Hence, continuous surveillance and effective antimicrobial stewardship programs are essential in the dairy industry to monitor and regulate antimicrobial use in veterinary practices. Future studies should also investigate the mechanisms contributing to AMR development and spread within environmental contexts. Additionally, studies should focus on utilizing whole-genome sequencing to analyze ARGs and phylogenetic relationships, exploring the connections between the environment, humans, and animals. These efforts are pivotal to proactively addressing One Health framework-related issues.

## Figures and Tables

**Table 1:** Prevalence of *E. coli* from dairy farms based on various samples.

Farms	No. of Samples Collected (*n*)	No. of Positive *E. coli*/Total No. of Samples (%)			
		Soil	Effluent	Cow Dung	Milk
1	24	3/6 (50.0)	2/6 (33.3)	4/6 (66.7)	2/6 (33.3)
2	24	2/6 (33.3)	4/6 (66.7)	5/6 (83.3)	2/6 (33.3)
3	24	4/6 (66.7)	4/6 (66.7)	6/6 (100.0)	2/6 (33.3)
4	24	5/6 (83.3)	2/6 (33.3)	4/6 (66.7)	4/6 (66.7)
5	24	3/6 (50.0)	5/6 (83.3)	5/6 (83.3)	3/6 (50.0)
6	24	4/6 (66.7)	5/6 (83.3)	6/6 (100.0)	6/6 (100.0)
7	24	4/6 (66.7)	4/6 (66.7)	5/6 (83.3)	3/6 (50.0)
8	24	3/6 (50.0)	5/6 (83.3)	6/6 (100.0)	5/6 (83.3)
Total	192	28/48 (58.3)	31/48 (64.6)	41/48 (85.4)	27/48 (56.3)

**Table 2:** AMR profiling of the *E. coli* isolates.

Antimicrobial Resistance Profile ^a^	Number of Antimicrobial Categories	Isolates, *n*
AMP AMC AMS CFE CPA CFN CEFO CEFX GEN CIP TMP	8	1
AMP AMS CFE CPA CEFO CEFZ CEFX CEFE CIP TMP	6	1
AMP AMC CFE CPA CFN TMP	5	1
AMP CFE CPA CFN CEFO CEFX CEFE	4	1
AMP CFE CPA CEFO CEFX GEN	4	2
AMP AMS CIP TMP	4	7
AMP GEN CIP TMP	4	1
AMP CFE CPA CEFO CEFX CEFE	3	1
AMP CFE CPA CEFO CEFX	3	3
AMP AMC AMS TZP	3	1
AMP AMC TMP	3	2
AMP AMS TMP	3	5
AMP AMS CIP	3	3
AMP CIP TMP	3	1
AMP GEN CIP	3	1
AMP AMS GEN	3	1

^a^ AMP, ampicillin; AMC, amoxicillin-clavulanic acid; AMS, ampicillin–sulbactam; CFE, cefuroxime; CPA, cefuroxime axetil; CFN, cefoxitin; CEFO, cefotaxime; CEFZ, ceftazidime; CEFX, ceftriaxone; CEFE, cefepime; GEN, gentamicin; CIP, ciprofloxacin; TMP, trimethoprim–sulfamethoxazole; TZP, piperacillin–tazobactam.

**Table 3:** AMR profiling of the ESBL-producing isolates.

ESBL-Producing Isolates	Antimicrobial Resistance Profile ^a^
Cow dung	
Isolate 1	AMP, CFE, CPA, CEFO, CEFX, GEN
Isolate 2	AMP, CFE, CPA, CEFO, CEFX, GEN
Isolate 3	AMP, CFE, CPA, CFN, CEFO, CEFX, CEFE
Isolate 4	AMP, CFE, CPA, CEFO, CEFX, CEFE
Milk	
Isolate 1	AMP, AMC, AMS, TZP
Isolate 2	AMP, CFE, CPA, CEFO, CEFX
Effluent	
Isolate 1	AMP, CFE, CPA, CEFO, CEFX
Isolate 2	AMP, AMS, CFE, CPA, CEFO, CEFZ, CEFX, CEFE, CIP, TMP

^a^ AMP, ampicillin; AMC, amoxicillin-clavulanic acid; AMS, ampicillin–sulbactam; CFE, cefuroxime; CPA, cefuroxime axetil; CFN, cefoxitin; CEFO, cefotaxime; CEFZ, ceftazidime; CEFX, ceftriaxone; CEFE, cefepime; GEN, gentamicin; CIP, ciprofloxacin; TMP, trimethoprim–sulfamethoxazole; TZP, piperacillin–tazobactam.

## Data Availability

The datasets used and/or analyzed during the current study are available in the NIH-DaRS repository or from the corresponding author upon reasonable request.

## Abbreviations

The following abbreviations are used in this manuscript:

AMR	antimicrobial resistance
*E. coli*	*Escherichia coli*
ARB	antimicrobial-resistant bacteria
MDR	multidrug resistance
ARGs	antimicrobial resistance genes
DVS	Department of Veterinary Services
NPRA	National Pharmaceutical Regulatory Agency
CLSI	Clinical Laboratory Standards Institute
EUCAST	European Committee on Antimicrobial Susceptibility Testing
ESBL	extended spectrum beta-lactamase
MICs	minimum inhibitory concentrations
